# Brain-Inspired Domain-Incremental Adaptive Detection for Autonomous Driving

**DOI:** 10.3389/fnbot.2022.916808

**Published:** 2022-06-15

**Authors:** Weihao Liang, Lu Gan, Pengfei Wang, Wei Meng

**Affiliations:** ^1^Guangdong Provincial Key Laboratory of Intelligent Decision and Cooperative Control, School of Automation, Guangdong University of Technology, Guangzhou, China; ^2^Robotics and Autonomous System, System Hub, The Hong Kong University of Science and Technology (Guangzhou), Guangzhou, China; ^3^Temasek Laboratories, National University of Singapore, Singapore, Singapore

**Keywords:** unsupervised domain adaptation, incremental learning, object detection, autonomous driving, domain incremental detection

## Abstract

Most existing methods for unsupervised domain adaptation (UDA) only involve two domains, i.e., source domain and the target domain. However, such trained adaptive models have poor performance when applied to a new domain without learning. Moreover, using UDA methods to adapt from the source domain to the new domains will lead to catastrophic forgetting of the previous target domain. To handle these issues, inspired by the ability to balance the maintenance of old knowledge and learning new knowledge of the human brain, in this article, we propose a new incremental learning framework for domain-incremental cases, which can harmonize the memorability and discriminability of the existing and the novel domains. By this means, the model can imitate the learning process of the human brain and, thus, improve its adaptability. To evaluate the effectiveness of the proposed methods, we conduct two groups of experiments, including virtual-to-real and diverse-weather cases. The experimental results demonstrate that our approach can avoid catastrophic forgetting, mitigate performance degradation in the previous domains, and improve the object detection accuracy of the novel target domain significantly.

## 1. Introduction

The safety of autonomous driving depends on the perceptual models of self-driving cars. With the detection results, the vehicles can plan a reasonable trajectory to avoid traffic accidents. In which, object detection is a fundamental and essential task for autonomous driving. It is similar to humans walking on the streets and crossing the roads. Each person has a learning mechanism to observe the positions of incoming cars and pedestrians. When building self-driving vehicles, to improve the robustness of the object detection algorithms, researchers used to train them with numerous labeled datasets containing as many situations as possible, which have achieved quite promising results. However, the relevant disadvantages are also prominent. For example, dataset annotation is expensive and requires a huge burden of work, i.e., the KITTI dataset includes 15,000 images containing over 80,000 objects. Moreover, it is well known that different datasets have different data distributions. For example, the data distribution of the Rainy-Cityscapes dataset is different from the typical Cityscapes dataset because of the raindrop imprint imposed. Therefore, in terms of the practical applications, Domain adaption becomes a promising research direction to overcome these problems by transferring knowledge from the unlabeled (the source domain) to the labeled (the target domain) data.

The actual driving scenarios are complex and various. But the aforementioned existing domain-adaption methods can only adapt to two scenarios (one labeled and one unlabeled) and is difficult to cover all possible cases, which differs from the learning mechanisms of the human brain. The brains are capable to learn all cases progressively to deal with all kinds of emergency situations without forgetting previous cases. Moreover, the original purpose of building a self-driving vehicle is to endow a normal car with the intelligence of human brains. Thus, improving the generalization capability of domain adaption models is key to solving this issue. One possible way is to train a model for each target domain and select the most suitable one based on predefined rules. However, normally the size of the model parameters is huge and could cost much computation resources for the parameter storage, loading, and switching. Another way is to simply re-train the model by using another target domain, which may lead to a forgetting issue of the previously learned domains. To solve these problems, in this study, we propose that all target domains should be conducted sequentially instead of simultaneously so that they cannot be blended into a whole during the training process of the detectors.

**Figure 1:**
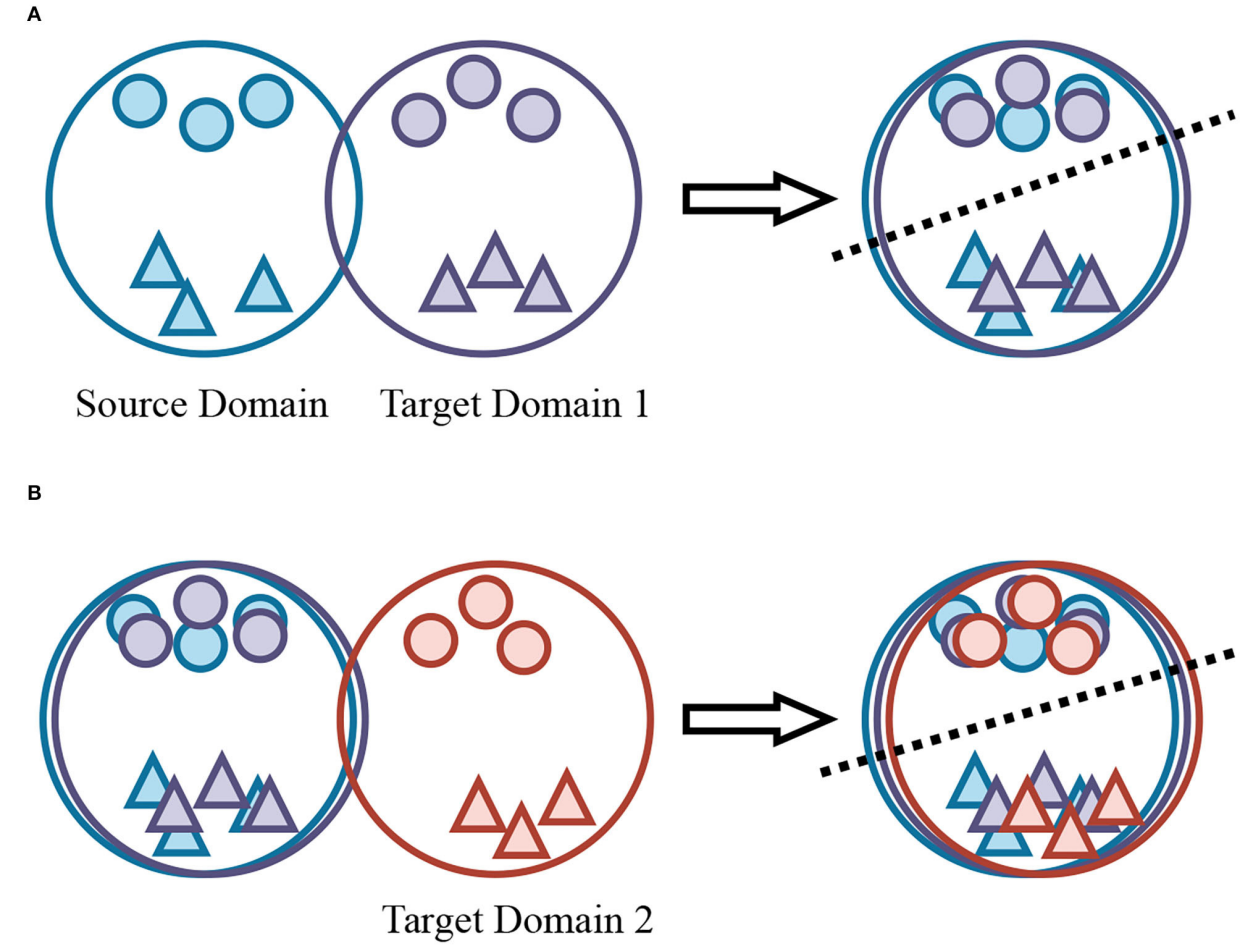
Illustration of the single-step domain adaptation and domain-incremental adaptation. The color blue, purple, and red represents the source domain, the first target domain **(A)**, and the second target domain **(B)**, respectively, while different shapes mean different classes in one domain. Single-step domain adaptation consists of pixel-level and instance-level alignment to further minimize the domain gap. For domain-incremental cases, the novel target domain needs to align with all previous domains.

To this end, as shown in Figure 1 we introduce incremental learning which is inspired by the characteristics of the human brain in progressive learning and continuous learning. Taking into consideration of the domain adaption challenges in complex autonomous driving scenarios, this article proposes a novel domain-incremental adaptive detection framework that can continually make the model adapt from one domain to another at multiple levels. It is as though humans can develop the adaptation to a new environment. The incremental-learning model learns to complete “tasks” one by one, where “tasks” refers to one step of unsupervised domain adaptation. By using the proposed framework, we extend the single-step adaptation to domain-incremental cases. Particularly, the incremental dataset contains previous and new parts. The former consists of a labeled source domain and several unlabeled target domains, while the latter is usually another unlabeled target domain without training. The proposed framework aims to maintain the memorability of previous domains and enhance the detectability of the new domains. One challenge behind is how to form a new task, i.e., deciding which old domain (the source domain or one of the past target domains) to adapt to the new one (the target domain). To tackle this problem, our strategy is to find divergences between the new target domain and all previous domains through dimensionality reduction and select the smallest one of them. Thus, our domain-incremental learning framework can be divided into two stages, i.e., “Recall” and “Adapt” stages. The “Recall” stage recalls the knowledge of old target domains through performing adaptation from the source domain and the latest target domain. To reduce the adverse effects of incorrect pseudo labels, we introduce “Domain-Mix” to combine it with the ground-truth labels of the source domain and extend it with patch-based adversarial learning, better integrating the two domains as one domain. While the “Adapt” stage learns the transfer of knowledge given the pseudo labels from the last target domain. In every step of the “Adapt” stage, the model is fed by an image that contains information about the two domains, then views them as a new source domain, and finally adapts toward the new target domain. Both two stages are indispensable because the “Recall” stage can generate more accurate pseudo labels of the previous target domain, playing a foundational role in the “Adapt” stage. The experiment results will prove this point.

**The highlight of the contributions**: We discuss why general incremental learning cannot apply to multiple domain scenes. After analyzing the research gap in the existing methods, we propose a domain-incremental learning framework and design a domain tree to decide the domain adaptation order. In the framework, we introduce “Domain-Mix” and design patch-based adversarial learning to refine the quality of pseudo labels, thus enhancing the discriminability on two domains without extra computational cost. Experiments and comparison results demonstrate that our approach achieves the best performance in domain-incremental adaptive object detection problems. To the best of our knowledge, we are the first of reporting incremental adaptation results from a virtual domain to multiple actual domains.

The remainder of the article is organized as follows. Section 2 briefly introduces some related studies. In Section 3, we address the problem formulation and single-step domain adaption method. In Section 4, we present the proposed domain-incremental adaptation algorithm. Experimental results are shown in Section 5. Section 6 concludes the article.

## 2. Related Study

### 2.1. Unsupervised Domain Adaptation for Object Detection

The key idea of adversarial learning (Chen et al., 2018, 2020; Saito et al., 2019; Csaba et al., 2021; Vibashan et al., 2021) is forcing backbone networks to produce domain-invariant features, which is useful for detecting target domains, and confusing domain discriminators by using a Gradient Reversal Layer (GRL) module. In Khodabandeh et al. (2019), Kim S. et al. (2019), Zhao et al. (2020), and Csaba et al. (2021), the authors utilize high-confident results of the target domain by a source-trained model and re-trained it on the target model. Due to the domain discrepancy existing between the source and the target domains, the authors translate target-domain images into source-like ones, commonly using Generated Adversarial Networks (GANs) (Chen et al., 2020; Hsu et al., 2020; Csaba et al., 2021). Contrary to the former, domain randomization (He and Zhang, 2019; Kim T. et al., 2019) is to translate the source domain into target-like images for generalization on the target domain. Mean-Teacher (Cai et al., 2019; Deng et al., 2021) is similar to self-supervised learning on unlabeled data, transferring knowledge from a source-teacher model to a target-student model. Objective relations can be modeled by graphs and limited *via* regularization (Cai et al., 2019; Xu et al., 2020) for detection.

Most recent studies use GAN-based approaches, such as Chen et al. (2020) and Csaba et al. (2021). However, these approaches are not applicable for domain-incremental adaptation because GANs aim at only a pair of domains per time. In incremental settings, the number of domains is normally more than two. Thus, GANs should be re-trained among previous domains and new domains, which is time and labor-consuming for multi-scenario deployment.

### 2.2. Incremental Learning

Incremental learning is also known as continual learning or lifelong learning, which is proposed for dealing with catastrophic forgetting problems on previously learned tasks. As mentioned in Jing et al. (2022), on the one hand, the algorithm is required to integrate new knowledge and transfer old knowledge (plasticity). On the other hand, it must prevent the significant interference of new knowledge with existing knowledge (stability). Correspondingly, the human brain can keep old knowledge in mind and simultaneously extract the useful part to learn new knowledge. Therefore, the mechanism for balancing between plasticity and stability in brains inspires the study of incremental learning.

Existing studies mainly focus on classification tasks, which can be divided into three groups, i.e., rehearsal-based, regularization-based, and parameter-isolation-based methods. Rehearsal-based methods, like the meaning of “rehearsal,” are to hold a few data of historic tasks (Rebuffi et al., 2017; De Lange and Tuytelaars, 2020) or to generate it with a given data distribution (Lavda et al., 2018). Regularization-based methods include two sides: data-focused (Zhang et al., 2020; Kurmi et al., 2021) and prior-focused (Lee et al., 2017; Aljundi et al., 2018). The former mainly distills knowledge from previous-trained models to fit the new data, while the latter limits the variation of important model parameters. Parameter-isolation-based methods, namely different parameters for different tasks, copy (Xu and Zhu, 2018; Rajasegaran et al., 2020) or freeze (Mallya et al., 2018; Serra et al., 2018) old model parameters when meeting new tasks.

As mentioned before, the above existing methods are primarily for classification tasks, and only a few of them can be used for object detection tasks. Detection approaches mainly follow the framework of knowledge distillation (Ramakrishnan et al., 2020; Zhou et al., 2020) and meta-learning (Joseph et al., 2020). Both of them concentrate on class-incremental scenarios. However, domains and tasks in this study are synchronously incremental while the category space is shared. Liu et al. (2020) have proposed incremental methods across multiple datasets, transcending previous studies with only single-style datasets. But our study supposes that domain-gap is the main factor.

### 2.3. Incremental Multi-Domain Adaptation

Models can prevent forgetting previous domains by applying incremental learning to multi-domain adaptation. In Su et al. (2020), the authors utilize gradient regularization to hold discrimination of source domains and maintain that of the previous target domain. Similarly, Volpi et al. (2021) propose a domain randomization method for random domain distribution and design a meta-learning-based strategy for adapting to each auxiliary domain. In Kim et al. (2020), the authors propose to train a memory module for each target domain by Double Hinge Adversarial Loss. Wei et al. (2020) introduce a knowledge distillation term to ensure semantic-level consistency between the source domain and each target domain. This study consists of two same models, one for ensuring the consistency of the high-level semantic information, and the other for performing adversarial learning between the source domain and all target domains. It seems like a “multi-target domain adaptation.” An image from the source domain is constantly fed into two models, which requires much heavy computation. The different point of our study in this article is that we assume tasks and domains are both incrementally appearing while preferable pseudo-labels of one-task target domain can be utilized for training in the next task.

## 3. Single-Step Domain Adaptation

First, we introduce single-step domain adaption which is the base network for our proposed framework. Pixel-level and instance-level adversarial training strategies are adopted in this study to access domain-invariant features.

In single-step domain adaptation, it assumes that there exist two domains, one is a fully-annotated source domain $D^{S}={\left\{x_{i}^{S},y_{i}^{S}\right\}}_{i=1}^{n^{S}}$ where $x_{i}^{S}\in X^{S},y_{i}^{S}\in Y^{S}$ and another one is a raw target domain $D^{T}={\left\{x_{i}^{T}\right\}}_{i=1}^{n^{T}}$ where $x_{i}^{T}\in X^{T}$. According to the definition of unsupervised domain adaptation, these two domains have different data distributions but share the same categories. That is to say, $P\left(X^{S}\right)\neq P\left(X^{T}\right)$ and $C\left(X^{S}\right)=C\left(X^{T}\right)$, where P and C indicate the data distribution and category space, respectively.

### 3.1. Multi-Level Domain Adaptation

**Figure 2:**
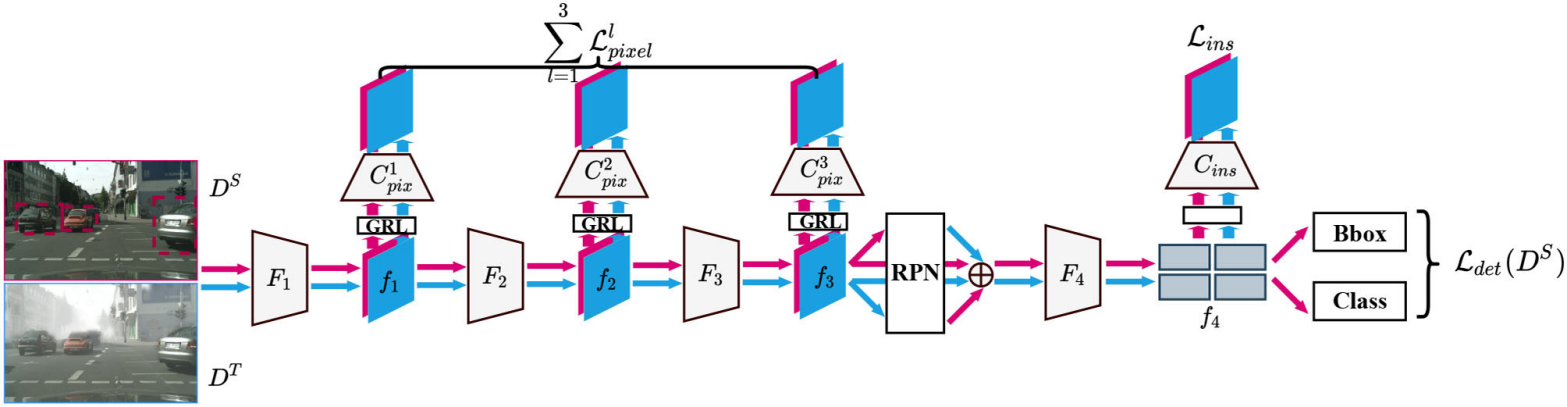
Overview of single-step domain adaptive model. The pink color represents the source domain, while the blue represents the target. This structure has three pixel-level domain discriminators ($C_{pix}^{1}$ to $C_{pix}^{3}$) and one instance-level discriminator (*C*_*ins*_).

As shown in Figure 2, the backbone network can be separated into three parts *F*^*l*^(*l* = 1, 2, 3), to acquire three-level features *f*^*l*^ for pixel-level adaptation. Besides, we denote the two fully-connected layers after the ROI-Align module as *F*^4^ and the output of *F*^4^ as *f*^4^ for instance-level adaptation. Before adaptation, all of the *f*^*l*^(*l* = 1, 2, 3, 4) pass Gradient Reversal Layers (GRLs) for extracting domain-invariant features by using adversarial training. Three pixel-level domain classifiers $C_{pix}^{l}$ and one instance-level domain classifier *C*_*ins*_ with a fully-convolutional structure, are constructed to discriminate which domain each pixel (instance) of the features *f*^*l*^ is from. The optimization objectives of four domain classifiers are to output corresponding domain maps, 0 for the source domain and 1 for the target domain.


(1) $$\begin{array}{r} L_{pixel}^{l}=L_{pixel}\left(C_{pix}^{l}\left(f_{S}^{l}\right),0\right)+L_{pixel}\left(C_{pix}^{l}\left(f_{T}^{l}\right),1\right) \end{array}$$

(2) $$\begin{array}{r} L_{ins}=L_{ins}\left(C_{ins}\left(f_{S}^{4}\right),0\right)+L_{ins}\left(C_{ins}\left(f_{T}^{4}\right),1\right) \end{array}$$

where $L_{pixel}$ and $L_{ins}$ are regarded as cross-entropy and focal loss, respectively. $f_{S}^{l}$ and $f_{T}^{l}$ represent the *l*-th layer features from source domain data $D^{S}$ and target domain $D^{T}$, respectively.

### 3.2. Single-Step Objective Function

Denote *C* and *B* as the abbreviation of all domain classifiers and remaining networks, respectively. Combining supervised loss $L_{det}$ and unsupervised domain adaptation loss $L_{adv}$ with a trade-off weight λ, the overall loss function can be designed as


(3) $$\begin{array}{r} L_{adv}\left(D^{S},D^{T}\right)=\overset{3}{\underset{l=1}{\sum }}L_{pixel}^{l}+L_{ins} \end{array}$$

(4) $$\begin{array}{r} L_{all}\left(D^{S},D^{T}\right)=L_{det}\left(D^{S}\right)-\lambda \cdot L_{adv}\left(D^{S},D^{T}\right) \end{array}$$

Because only the source images have ground-truth labels, the supervised loss is only performed on the source domain $D^{S}$. Moreover, the unsupervised adversarial loss is adopted in two domains, $D^{S}$ and $D^{T}$. The subtraction sign represents adversarial learning.


(5) $$\begin{array}{r} M\leftarrow \underset{B}{\min}\underset{C}{\max}L_{all}\left(D^{S},D^{T}\right) \end{array}$$

Given the loss function shown in Equation 4, the optimization objective is to maximize the gradient from $L_{adv}$ of domain classifier *C* and minimize that of other modules *B*. In this way, *B* aims to confuse the discrimination of *C* and generate domain-invariant features. Thus, we can obtain the adapted model *M* from the source domain $D^{S}$ to the target domain $D^{T}$.

## 4. Domain-Incremental Adaption

Based on the single-step domain adaption in Section 3, the framework of domain-incremental adaption can be further constructed in this section. To overcome the computation intensive and under-fitting problems, different from the traditional methods which either train a model for each pair of source-target domains or connect target domains in one, we propose a domain-incremental adaptation framework that adapts from a mixed source domain to the new target domain. We refer to the process of the human learning, dividing the whole incremental learning framework into two main parts: the “Recall” and “Adapt” stages. The former is akin to reviewing the previously learned knowledge (previous domains). The latter is similar to studying new knowledge (the novel target domain), which is performed by finding the common point between the old knowledge and the new knowledge. After training through the proposed framework, the final model can maintain a balance between the memorability of previous domains and the discriminability of the new domains. Details will be given in this section.

First, we provide some preliminaries. Compared with single-step domain adaptation, domain-incremental adaptation contains multiple unlabeled target domains instead of one, i.e., $D_{k}^{T}={\left\{x_{i}^{T}\right\}}_{i=1}^{n^{T}}$. In contrast to multi-target domain adaptation, domain-incremental adaptation is required to learn step-by-step, i.e., first from the labeled source domain $D^{S}$ to the first unlabeled target domain $D_{1}^{T}$, then to the second unlabeled target domain $D_{2}^{T}$. The relationship between each target domain and the source domain, without doubt, follows the principle above. Moreover, all the target domains have mutually unique data distributions but shared semantic space, namely, $P\left(X_{m}^{T}\right)\neq P\left(X_{n}^{T}\right)$ and $C\left(X_{m}^{T}\right)=C\left(X_{n}^{T}\right)$ where *m*≠*n*.

### 4.1. Build Domain Tree

In single-step domain adaptation, which only adapts from one domain $D^{S}$ to another $D_{1}^{T}$, the adaptation order is determined. In contrast to the single-step adaptation, domain-incremental adaptation is supposed to continually adapt toward a new target domain $D_{2}^{T}$ after performing its latest adaptation process. The model needs to build the next adaptation task in order to transfer knowledge from the previous domains to the new one. Specifically, it has two options: either from $D^{S}$ to $D_{2}^{T}$ or from $D_{1}^{T}$ to $D_{2}^{T}$, which depends on the discrepancy between the two domains. If the domain discrepancy between $D_{1}^{T}$ and $D_{2}^{T}$ is smaller than that of between $D^{S}$ and $D_{2}^{T}$, we ought to design the next task from $D_{1}^{T}$ to $D_{2}^{T}$ because a smaller domain discrepancy contributes to easier knowledge transfer.

**Figure 3:**
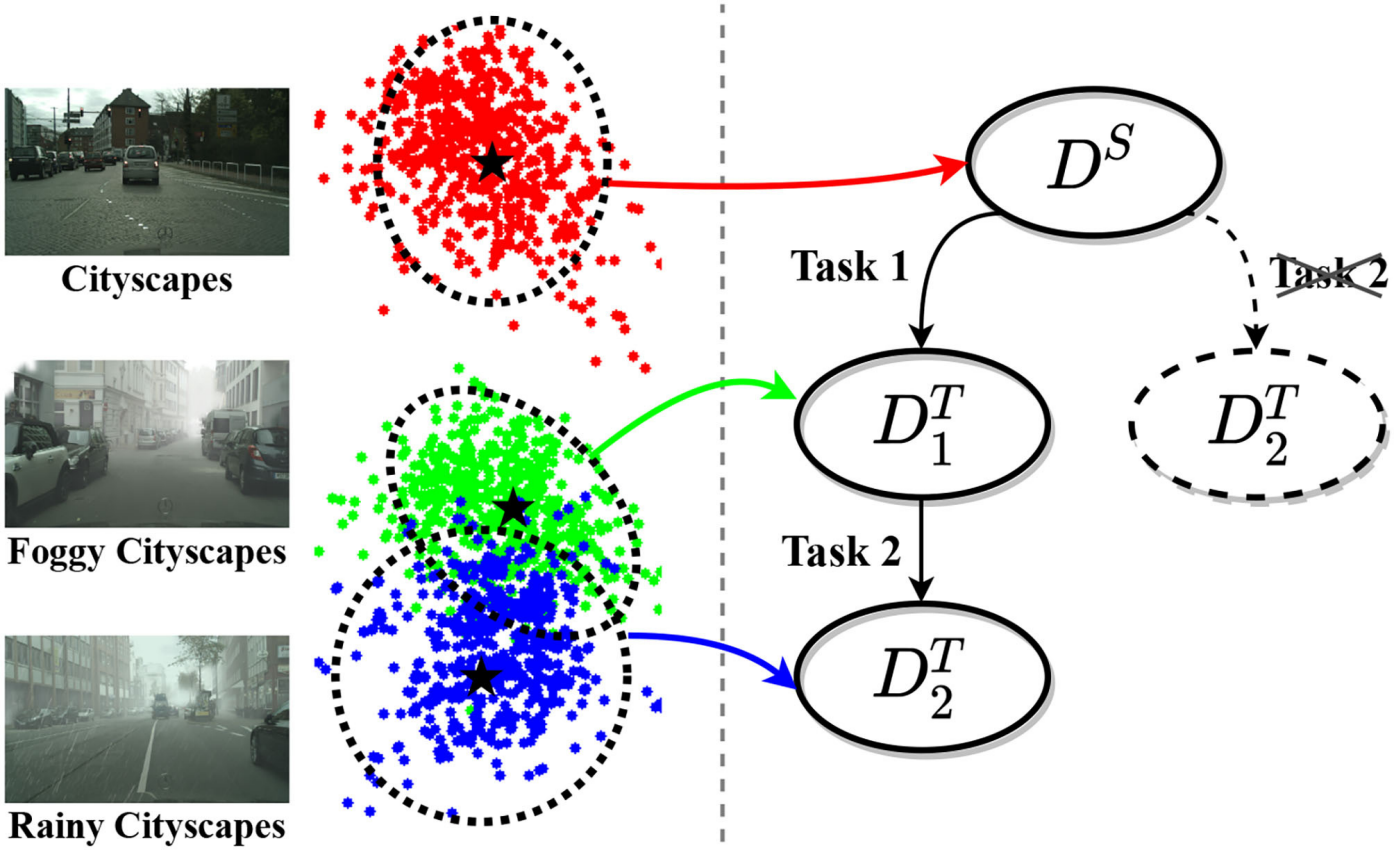
Left: Visualization of data distributions of Cityscapes (Cordts et al., 2016), Foggy-Cityscapes (Sakaridis et al., 2018), and Rainy-Cityscapes (Hu et al., 2019), indicated by red, green, and blue dots, respectively. Right: Illustration of Domain Tree.

Here, we provide a simple example for illustration purposes. Assume that Cityscapes and Foggy-Cityscapes are the source domain $D^{S}$ and the first target domain $D_{1}^{T}$, respectively, a model adapting from Cityscapes to Foggy-Cityscapes is trained, namely “Task 1.” In the following, Rainy-Cityscapes appears in the form of a new target domain $D_{2}^{T}$. To ascertain the discrepancies among these three domains, we sample an equal number of images from each domain at random and adopt approaches for data dimension reduction (Van der Maaten and Hinton, 2008). As shown in Figure 3, the center of each domain is calculated and marked with a star. It is clear to find that the discrepancy between Foggy-Cityscapes $\left(D_{1}^{T}\right)$ and Rainy-Cityscapes $\left(D_{2}^{T}\right)$ is smaller than that between Cityscapes $\left(D^{S}\right)$ and Rainy-Cityscapes $\left(D_{2}^{T}\right)$. Therefore, the model is required to adapt from Foggy CitysScapes $\left(D_{1}^{T}\right)$ to Rainy-Cityscapes $\left(D_{2}^{T}\right)$ rather than from Cityscapes $\left(D^{S}\right)$ to Rainy-CitysScapes $\left(D_{2}^{T}\right)$, namely “Task 2.”

### 4.2. A Framework for Domain-Incremental Adaptation

#### 4.2.1. How to Perform Next Adaptation Task

Suppose that the model *M*_1_ completes the first domain adaptation task from $D^{S}$ and $D_{1}^{T}$ and is able to generate pseudo labels of the $D_{1}^{T}$. After determining the adaptation order during the whole domain-incremental learning process, we first consider that the model *M*_1_ can be continually adapted from $D_{1}^{T}$ to $D_{2}^{T}$. To be specific, $D_{1}^{T}$ acts as the source domain of the second domain adaptation task. The training steps are formulated as:


(6) $$\begin{array}{r} M_{1}\leftarrow \underset{B_{1}}{\min}\underset{C_{1}}{\max}L_{all}\left(D^{S},D_{1}^{T}\right) \end{array}$$

(7) $$\begin{array}{r} M_{2}\leftarrow \underset{B_{2}}{\min}\underset{C_{2}}{\max}L_{all}\left(D_{1}^{T},D_{2}^{T}\right) \end{array}$$

However, the above approach is susceptible to the inaccurate labels in $D_{1}^{T}$. We involve the source domain $D^{S}$ in the domain-incremental learning process in view of the ground-truth labels in $D^{S}$. Contrast to Wei et al. (2020) which feeds $D^{S}$ for knowledge distillation, we combine $D^{S}$ and $D_{1}^{T}$ as a new source domain $D_{new}^{S}$ for the second adaptation task. Therefore, the domain-incremental learning procedure can be reformulated as:


(8) $$\begin{array}{r} M_{1}\leftarrow \underset{B_{1}}{\min}\underset{C_{1}}{\max}L_{all}\left(D^{S},D_{1}^{T}\right) \end{array}$$

(9) $$\begin{array}{r} M_{2}\leftarrow \underset{B_{2}}{\min}\underset{C_{2}}{\max}L_{all}\left(D_{new}^{S},D_{2}^{T}\right) \end{array}$$

#### 4.2.2. Self-Training With Two Domains

The disadvantage of simply combining two datasets (domains) into one is obvious. On the one hand, if one domain has ground-truth labels while the other only has unreliable pseudo labels, training the model with these inaccurate labels will decrease the performance of the latest target domain. On the other hand, sampling images randomly from a hybrid dataset can cause inconsistent data distribution of two consecutive inputs. This case will reduce the model generalization capabilities and increase the difficulties in model fitting, thus obtaining sub-optimal results.

**Figure 4:**
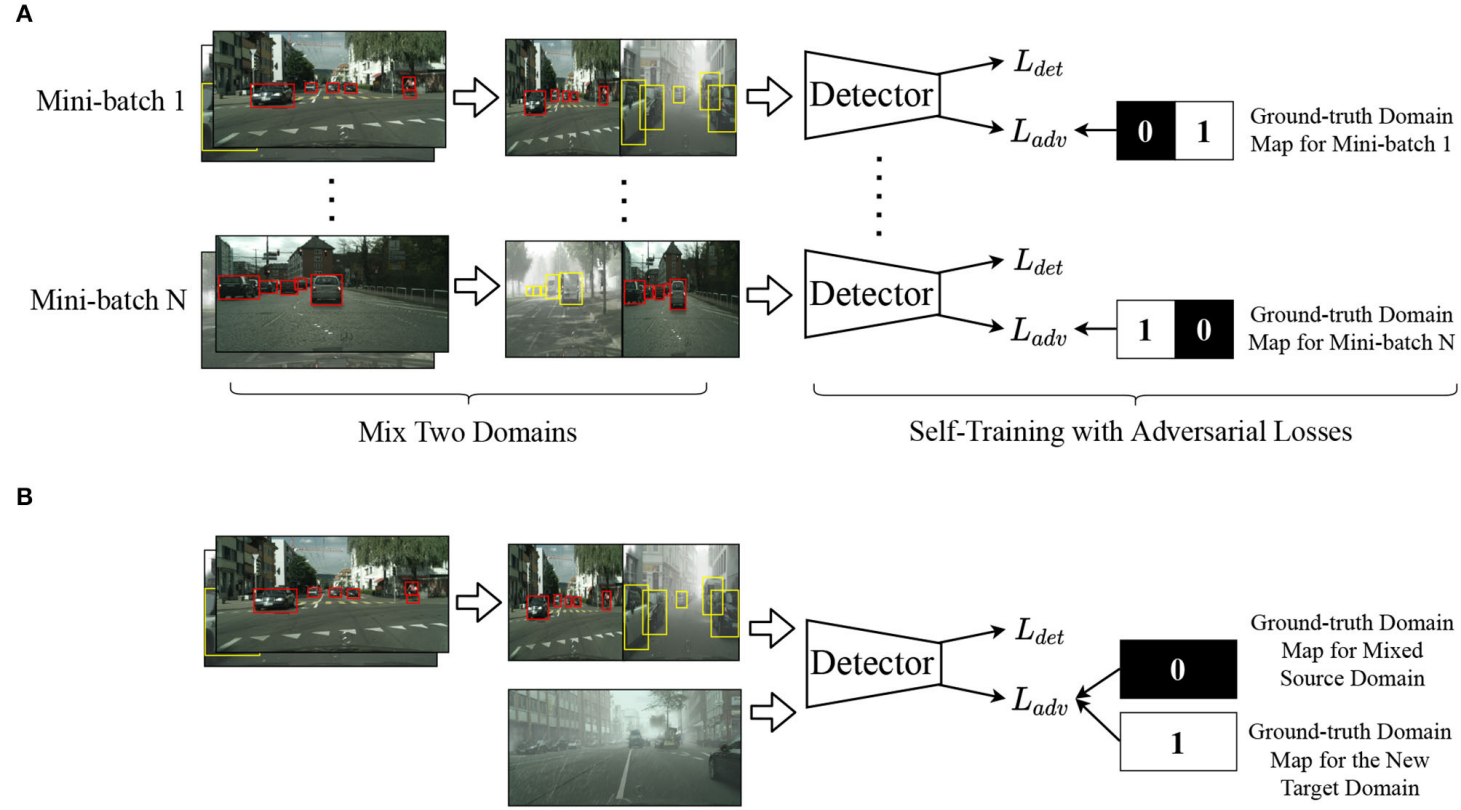
**(A,B)** Illustration of the “Recall” and “Adapt” stage. In the first “Recall” stage, boxes in red and yellow represent ground-truth labels of the source domain and pseudo labels of the latest target domain, respectively. In the second “Adapt” stage, we consider the mixed source domain as the source domain and complete adaptation. Black and white in the ground-truth domain maps signify the source domain (0) and the target domain (1).

To solve these problems, inspired by Ramamonjison et al. (2021), we propose to sample one image from the source domain $D^{S}$ and the latest target domain $D_{k}^{T}$, respectively, halve their long edges and assemble them from left to right in a stochastic order. We adopt similar ways to transform and concatenate corresponding data for annotations. Thus, a step of input can both contain images from two different domains and not bring extra computational costs. The shape of input images remains the same before combining two images and after processing. Moreover, we extend the self-training framework with patch-based adversarial losses. In pixel-level and instance-level domain adversarial learning, it usually takes a tensor of zeros or ones as the optimization goals of domain maps when the input is from either the source domain or the target domain. However, for our cases, we modify the ground-truth domain maps because an input image contains information from two domains. As shown in the upper right corner of Figure 4, if the left side of the input is from the source domain, the left half of the ground-truth domain map is composed of zeros or ones otherwise. The primary purpose is to enhance discriminability on two domains in one image. Batch Normalization (BN) layers (Wang et al., 2019) in a trained model are responsible to store the running mean and variance in a batch of images, encoding the style-specific information from each domain so that we only update the learnable parameters in BN layers while freezing other parameters during the self-training process. Given only one image, the model can generate domain-invariant features for both the source domain and the target domain through the domain discriminator. After performing self-training with an adapted model, the quality of pseudo labels of $D_{k}^{T}$ is further enhanced and we can fetch refined pseudo labels for the following domain adaptation task.

#### 4.2.3. Domain-Incremental Learning

On the basis of the aforesaid “Self-Training” strategy, we propose a two-stage domain-incremental learning framework. Suppose a model trained from the source domain $D^{S}$ and the k-th target domain $D_{k}^{T}$ by using the single-step domain adaptation algorithm mentioned in Section 3 can be obtained as *M*_*k*_. Coarse pseudo labels of $D_{k}^{T}$ are generated with the weight of *M*_*k*_, coming into $D_{kp}^{T}$ where the subscript *p* represents a target domain with pseudo labels. In the first stage named “Recall,” we group $D^{S}$ and $D_{kp}^{T}$ into $D_{new}^{S}$, feed it into the network, and train *M*_*k*_ with the above “Self-Training” strategy. Stage “Recall” aims to raise the quality of pseudo labels and reduce the degree of knowledge forgetting, which will be further demonstrated in the later ablation study section. In the second stage “Adapt,” we update the labels of $D_{new}^{S}$ *via* the refined pseudo labels of $D_{kp}^{T}$ from the “Recall” stage. After that, we take $D_{new}^{S}$ and $D_{k+1}^{T}$ as the labeled source and unlabeled target domain respectively and perform a new domain adaptation task with both detection and adaptation losses. In this stage, the ground-truth domain map of $D_{new}^{S}$ is 0 instead of a combination of 0 and 1. Details of our proposed learning framework are shown in Figure 4 and Algorithm 1.

**Table:** Domain-incremental adaptation framework.

**Input:** Domain data *D*^*S*^, $D^{T}=\left\{D_{1}^{T},...,D_{K-1}^{T}\right\}$, $D_{K}^{T}$. **Output**: Final model *M*.
1: **if *K*=1 then**
2: ******** First domain adaptation task ********
3: Initialize model from scratch.
4: Perform domain adaptation with *D*^*S*^ and $D_{1}^{T}$.
5: Get adaptive model *M*_1_.
6: **else**
7: ******** Domain-incremental adaptation task ********
8: Initialize model from *M*_*K*−1_.
9: Generate coarse pseudo label from $D_{K-1}^{T}$.
10: Integrate domain data $D_{new}^{S}=\left\{D^{S},D_{K-1}^{T}\right\}$ *via* DomainMix.
11: “Recall Stage”: Self-Training with patch-based domain adaptation.
12: “Adapt stage”: Update labels of $D_{new}^{S}$ and perform Domain adaptation from $D_{new}^{S}$ to $D_{K}^{T}$.
13: Obtain adaptive model *M*_*K*_.
14: **end if**

## 5. Experiments

### 5.1. Datasets and Scenarios Setting

#### 5.1.1. Datasets

We utilize four commonly used datasets to verify the effectiveness of our proposed framework. **Sim10K** (Johnson-Roberson et al., 2017) is a virtual dataset consisting of 10,000 images snapped from Grand Theft Auto V (GTA5). Notably, it contains only one category (“Car”). **Cityscapes** (Cordts et al., 2016) is a common dataset with urban driving scenes from 50 different cities under clear weather and contains 2,975 training images and 500 validation images. On account of pixel-wise annotations, we utilize the minimum enclosing rectangle to obtain its bounding-box labels. **Foggy-Cityscapes** (Sakaridis et al., 2018) and **Rainy-Cityscapes** (Hu et al., 2019) are synthetic datasets that adopt GAN-like techniques to generate various degrees of foggy and rainy weather. Both Foggy-CitysScapes and Rainy-CitysScapes have identical content (annotations and subset split) with CitysScapes. Rainy-CitysScapes lacks the “train” class in the validation subset. Thus, we randomly sample 35 images from all 169 images with the “train” class in the training subset and incorporate them with the original validation subset.

#### 5.1.2. Group of Experiments

Given these datasets, we design two series of experiments to evaluate our model in domain-incremental adaptation: (i) **Diverse Weathers**. Cityscapes → Foggy-Cityscapes → Rainy-Cityscapes. The performance of all eight classes (bus, bicycle, car, motorcycle, person, rider, train, truck) is observed and the total precision is calculated. (ii) **Virtual-to-Real**. SIM10K → Cityscapes → Foggy-Cityscapes → Rainy-Cityscapes. Due to the category space limitation of the Sim10K, this series of experiments only validate the “Car” models.

### 5.2. Implementation Details

We build our detector with ResNet-101 (He et al., 2016) pre-trained on ImageNet (Russakovsky et al., 2015) datasets as the backbone network to extract accurate visual representations for subsequent domain adaptation. Each input image is resized to 600 pixels on the shorter side through the training process and pre-processed *via* random flipping. During each iteration of training, one source-domain image and one target-domain image are input successively. For the primary domain adaptation task, from the source domain to the first target domain, we follow a regular training strategy with a learning rate of 0.001 for 50k iterations and then decrease by a factor of 10 for the last 20k iterations. For the other adaptation tasks, e.g., from the first target domain to another new target domain, for instance, we maintain a lower learning rate of 0.0001 and report the model which performs best on the Rainy-Cityscapes dataset. When generating pseudo labels, we use a confidence threshold of 0.5 and 0.7 for Experiments Group I and II, respectively. At the validation phase, we demonstrate the performance of our model by the mean average precision (mAP) of all categories with a widely-used intersection over union (IoU) threshold of 0.5. Supposed that *AP*_*i*_ is the precision of category *i*, the mAP is calculated by the means of all *AP*_*i*_. Finally, we use the PyTorch framework to implement our domain-incremental learning framework.

### 5.3. Comparison Results With Incremental Learning Methods

Considering our domain-incremental learning framework, orthogonal to all single-step domain adaptation methods, we do not compare it with other state-of-the-art proposals in single-step settings. However, we also incorporate some of them into our framework to evaluate their effectiveness because the main focus of this article is incremental learning applied in domain adaptation, such as SWDA and HTCN without interpolation (hereinafter called HTCN).

We perform three different incremental learning strategies for each origin single-step domain adaptation method. For the sake of fairness, each comparison is conducted on the basis of the same single-step domain adaptation approach. The strategies are briefly described below. “MIX” means that the model is continually trained on mixed target domains, including old and new ones. “FT” is to directly fine-tune the model with the new domain as the target domain while keeping the source domain. Similarly, “PFT” firstly generates pseudo labels *via* trained models of the last adaptation task and then utilizes them to perform the next adaptation period. Besides, we also specify the lower-bound and upper-bound bounds. “Source only” trains a detector only with the source domains and evaluates it on target domains without adaptation to new target domains. Other than “PFT,” “SFT” fine-tune the last trained model from the old target domain with ground-truth labels to new domains, while “SMFT” has access to the ground-truth source and the last target domain. Moreover, “SSFT” also adopts a two-stage training strategy but utilizes ground-truth labels of the last target domain in the second stage. The degree of forgetting during the incremental learning process is marked *via* a number with the symbol “↓.” It is calculated by subtracting *MAP*_*pre*_ from *MAP*_*aft*_ where *MAP*_*pre*_ is the accuracy before adapting to the new target domain while *MAP*_*aft*_ means the precision after adapting. We choose “ILB” (Wei et al., 2020) as the state-of-the-art domain-incremental adaptation method. Due to a lack of source code, we reproduce “ILB” and report the results on our dataset settings.

#### 5.3.1. Diverse Weathers

In this section, we verify the performance of our proposed domain-incremental learning framework on “Diverse Weathers.” The first task is adapting from Cityscapes to Foggy-Cityscapes. The second task is continually adapting to Rainy-Cityscapes. First, we train a model with the single-step domain adaptation method described in Section 3, completing an adaptation task from Cityscapes to Foggy-Cityscapes. The detection results (37.1 on the Foggy-Cityscapes dataset) are shown as “Base-line” in Table 1. Then we report multiple comparison experiment results on both Foggy-Cityscapes and Rainy-Cityscapes. The former is to inquire about the degree of forgetting on the previous target domain, i.e., Foggy-Cityscapes, while the latter is to investigate the effects on the new target domain, i.e., Rainy-Cityscapes. Noted that all of the next experiments will be carried out on the basis of the “Base-line” model. The results are shown in Table 1. From the results, we can see that our method achieves the highest accuracy on the previous target domain (36.1 on Foggy-Cityscapes), obtaining a minimum performance decline (1.0 vs. 1.1 with PFT vs. 2.1 with MIX vs. 3.1 with FT vs. 1.5 with ILB). Meanwhile, our framework gets the highest accuracy on the new target domain (38.5 on Rainy-Cityscapes vs. 28.8 with Source-only vs. 32.5 with PFT vs. 36.5 with MIX vs. 37.7 with FT vs. 37.3 with ILB). It indicates that our approach has the ability to not only transfer knowledge from previous domains but also avoid the forgetting issue. By contrast, all other incremental learning methods have different levels of drawbacks. “FT” neglects to recall previous domains so that it is prone to cause catastrophic forgetting and a sharp decline in the performance of the previous target domain (from 37.1 to 34.0 on Foggy-Cityscapes) even though it gains a second-best result on the new target domain (37.7 on Rainy-Cityscapes). As for “MIX,” models achieve poor performance on both the previous and new target domain (35.0 on Foggy-Cityscapes and 36.5 on Rainy-Cityscapes). The main reason is that the model cannot adapt to diverse data distributions simultaneously. “PTF” can protect against loss of previous-learned knowledge (36.0 on Foggy-Cityscapes) but fails to learn new domains effectively (32.5 on Rainy-Cityscapes) due to incorrect pseudo labels. In addition, putting experiment results on Foggy-Cityscapes and Rainy-Cityscapes together, ILB obtains a suboptimal performance (35.6 on Foggy-Cityscapes and 37.3 on Rainy-Cityscapes). In summary, our proposed domain-incremental learning framework has the best comprehensive performance on whether previous or new target domain (36.1 on Foggy-Cityscapes and 38.5 on Rainy-Cityscapes). In the supervised methods, we adopt the ground-truth labels to replace pseudo labels and thus the performance has a large improvement. Moreover, “SMFT” has the highest accuracy, which proves that the combination of the source domain and the last target domain contributes to adapting to the new target domain.

**Table 1:** Results of “Diverse Weathers” adaptation.

**Methods**	**Bus**	**Bicycle**	**Car**	**Motorcycle**	**Person**	**Rider**	**Train**	**Truck**	**mAP**
**Foggy-Cityscapes**								
Source-only	32.1	31.6	36.0	24.1	25.9	39.4	9.1	16.4	26.8
Base-line	46.5	34.1	45.6	29.5	32.7	45.8	38.0	24.3	37.1
PFT	45.6	33.1	44.3	25.5	30.6	**44.8**	**41.3**	23.0	36.0(↓1.1)
MIX	44.3	33.2	43.7	**29.0**	29.8	42.2	34.1	23.7	35.0(↓2.1)
FT	46.7	31.9	43.8	25.8	28.8	43.4	34.3	25.1	34.0(↓3.1)
ILB	43.0	**34.5**	44.4	26.2	**31.5**	**44.8**	39.2	21.3	35.6(↓1.5)
**Ours**	**47.3**	34.3	**44.5**	25.9	31.1	44.2	35.2	**25.9**	**36.1**(↓1.0)
SFT	51.3	34.4	48.8	32.8	32.7	45.5	46.8	29.3	40.2
SMFT	52.4	36.3	45.7	33.8	33.3	46.2	45.7	29.6	40.4
SSFT	52.0	34.7	44.7	30.3	32.4	45.0	54.8	28.2	40.3
**Rainy-Cityscapes**							
Source-only	60.7	27.7	35.2	1.4	23.6	55.5	24.7	1.6	28.8
PFT	76.5	28.2	**44.2**	1.6	**24.4**	59.6	20.5	5.2	32.5
MIX	**97.3**	28.8	43.3	1.5	23.1	58.5	33.8	6.0	36.5
FT	95.3	27.8	43.7	2.2	23.8	59.7	**41.1**	8.4	37.7
ILB	91.6	31.4	44.1	**5.7**	24.1	**60.6**	33.1	7.9	37.3
**Ours**	89.1	**30.9**	44.0	1.7	23.6	60.2	38.7	**19.0**	**38.5**
SFT	87.4	32.4	48.7	3.4	25.1	60.8	50.2	25.7	41.7
SMFT	89.3	33.4	45.4	15.5	24.8	57.9	47.9	21.6	42.0
SSFT	89.3	28.0	44.2	6.7	23.7	56.9	50.2	20.9	40.0

*The bold and underline values represents the “Highest” and the “Second Highest” result*.

#### 5.3.2. Virtual-to-Real

**Figure 5:**
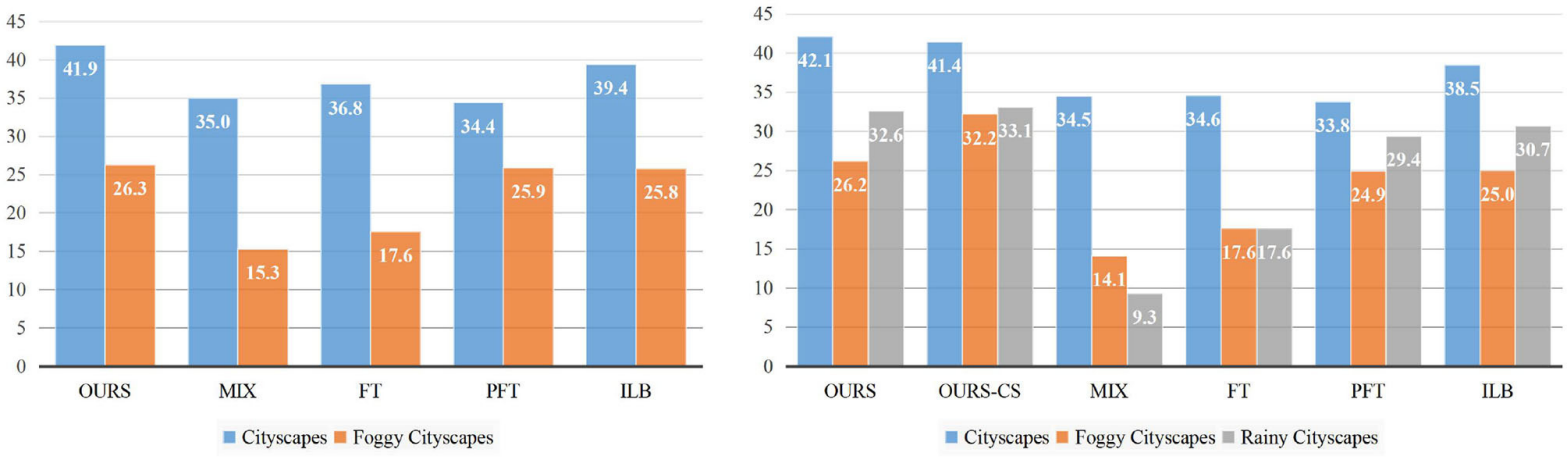
Left: The adaptation results from Sim10K to Foggy-Cityscapes. Right: The adaptation result from Sim10K to Rainy-Cityscapes. The vertical and horizontal axis represents overall performance (mAP) and different datasets (domains).

To the best of our knowledge, we are the first to survey incremental adaptation results from virtual datasets to real-world ones. In this section, the experiment includes three tasks: a) an initial task: adaptation from the source domain (Sim10K) to the first target domain (Cityscapes); b) the first incremental task: adaptation to the second target domain (Foggy-Cityscapes); c) the second incremental task: adaptation to the third target domain (Rainy-Cityscapes). On account of the increasing number of datasets, we display experimental results in the form of histograms rather than tables. Moreover, due to the availability of only one class in the source domain (Sim10K dataset), we only evaluate the performance of “Car.” The related experimental results can be found in Figure 5. Note that we do not draw the adaptation results on task a) but directly compare the accuracies on target domains after performing task b) and task c). This is because we can learn about the degree of forgetting on previous target domains by comparing the heights of blue bars (Cityscapes) and orange bars (Foggy-Cityscapes). The left side shows the results of task b). Our proposed domain-incremental learning framework gains the best performance (41.9 on Cityscapes and 26.3 on Foggy-Cityscapes) in comparison with other incremental learning based methods. For example, FT obtains a second-best result on the first target domain (36.8 on Cityscapes) but a poor performance on the second target domain (17.6 on Foggy-Cityscapes), which gets the same conclusion as demonstrated in Section 5.3.1. Other methods (MIX, PFT) have similar conclusions. Then we focus on the performance comparison of task c) shown on the right side of Figure 5. We perform domain-incremental adaptation experiments and train the model based on the trained model from task b). In terms of previous target domains, compared to the performance of task b), the accuracies show no significant decline (from 41.9 to 42.1 on Cityscapes and from 26.3 to 26.2 on Foggy-Cityscapes). In contrast, the performance declines significantly with the methods of whether MIX, FT, or PFT. The mAP of FT, for instance, drops from 36.8 to 34.6 on Cityscapes for being lack of constraints on previous target domains. With regards to the new target domain (Rainy-Cityscapes, signified by gray bars in Figure 5), our proposed domain-incremental adaptation framework obtains the best grade (32.6 vs. 9.3 with MIX vs. 17.6 with FT vs. 29.4 with PFT). Moreover, instead of directly utilizing the ground-truth labels of the source domain (Sim10K), we also attempt to generate pseudo labels of the first target domain (Cityscapes) to build task c). The result is shown as the “OURS-CS” bar on the right of Figure 5. Although the performance on Cityscapes slightly drops, the accuracies on Foggy and Rainy Cityscapes are actually improved, particularly for Foggy-Cityscapes (from 26.2 to 32.2). We suspect that the domain gap between the source domain (Sim10K) and the new target domain (Rainy-Cityscapes) contains not only a style-based gap but also a weather-based gap. Such a domain gap is too large for models to fit in, limiting the overall performance. This conclusion confirms our views in Section 4.1, and it is essential for domain-incremental learning to determine the adaptation order.

### 5.4. Ablation Study

#### 5.4.1. Two-Stage Training Strategy

To verify the impact of the “Recall” stage, when we obtain model *M*_*k*−1_ adapted from the source domain *D*^*S*^ to the last target domain $D_{k-1}^{T}$, we directly generate pseudo labels of $D_{1}^{T}$, combine it with *D*^*S*^ as a new source domain and adapt toward $D_{k}^{T}$. In Table 2, the sharp performance declines (2.6 and 3.5 on Foggy-Cityscapes and Rainy-Cityscapes, respectively) show that mere single-stage adapting from a mixed source domain to the new target domain degrades the performance on both previous and new target domains. It thereby proves that two stages play an integral role in further avoiding catastrophic forgetting and utilizing the ground-truth source domain to support the transfer of knowledge. Moreover, in the last line of the Table 2, we also compare the results with “Domain-Mix” or without “Domain-Mix.” The comparison results (degrading 1.9 and 2.7 on Foggy-Cityscapes and Rainy-Cityscapes) indicate that “Domain-Mix” contributes to generating refined pseudo labels of the last target domain. Without “Domain-Mix,” the training process of the “Adapt” stage can only adopt coarse pseudo labels with a tremendous amount of noise.

**Table 2:** Ablation study results on “Diverse Weathers” adaptation.

**Methods**	**Bus**	**Bicycle**	**Car**	**Motorcycle**	**Person**	**Rider**	**Train**	**Truck**	**mAP**
**Foggy-Cityscapes**								
HTCN-Baseline	46.2	35.4	44.3	25.6	32.3	46.5	35.1	26.7	36.5
HTCN-MIX	46.4	30.9	43.1	**27.7**	29.0	43.6	32.2	22.4	34.4(↓2.1)
HTCN-FT	41.7	29.0	42.8	23.4	27.8	40.2	33.2	26.8	33.1(↓3.4)
HTCN-PFT	47.5	**32.7**	**43.9**	23.4	**30.9**	**45.4**	**34.3**	27.4	**35.7**(↓0.8)
HTCN-ILB	47.6	31.8	43.4	24.2	29.5	44.9	26.7	29.3	34.7(↓1.8)
HTCN-Ours	**49.5**	32.0	43.6	24.3	30.0	43.9	31.1	**29.8**	35.5(↓1.0)
Ours	47.3	34.3	44.5	25.9	31.1	44.2	35.2	25.9	36.1
w/o Recall	43.7	35.4	42.5	22.1	28.8	41.2	34.4	23.8	33.5(↓2.6)
w/o Domain-Mix	44.0	31.9	41.8	25.6	28.2	42.6	33.1	26.2	34.2(↓1.9)
**Rainy-Cityscapes**								
HTCN-MIX	82.7	26.8	43.5	2.3	23.2	53.6	**41.2**	16.9	36.3
HTCN-FT	84.1	26.1	43.3	3.5	23.5	54.3	37.8	20.6	36.7
HTCN-PFT	76.4	27.3	**43.9**	3.9	**24.5**	**58.8**	33.2	18.2	35.8
HTCN-ILB	81.8	26.3	43.1	5.5	24.0	58.3	41.1	19.9	37.5
HTCN-Ours	**85.5**	**27.6**	43.5	**7.4**	23.5	58.5	37.5	**24.8**	**38.5**
Ours	89.1	30.9	44.0	1.7	23.6	60.2	38.7	19.0	38.5
w/o Recall	77.9	29.3	44.3	1.9	23.9	59.7	29.0	14.2	35.0(↓3.5)
w/o Domain-Mix	79.0	30.4	43.8	2.4	23.1	58.9	31.6	17.5	35.8(↓2.7)

*The bold and underline values represents the “Highest” and the “Second Highest” result*.

#### 5.4.2. Applicability to Other Domain Adaptation Methods

We consider “HTCN” (Chen et al., 2020) without interpolation while other settings remain the same as in Chen et al. (2020). From Table 2, it can be observed that HTCN with our proposed domain-incremental framework has a comprehensive optimum performance (35.5 on Foggy-Cityscapes and 38.5 on Rainy-Cityscapes) than other incremental-learning approaches. Although PFT gains 35.7 on the previous target domain (Foggy-Cityscapes) which is a little better than our methods, it remains a really poor performance on the new target domain (35.8 on Rainy-Cityscapes) due to inaccurate pseudo labels from Foggy-Cityscapes. Generally, ILB obtains the second-best accuracy on two target domains (34.7 on Foggy-Cityscapes and 37.5 on Rainy-Cityscapes). In a word, our proposed domain-incremental learning framework can acquire a state-of-the-art accuracy, no matter what the single-step domain adaptation algorithm is adopted.

#### 5.4.3. Performance With Different Confidence Thresholds

**Figure 6:**
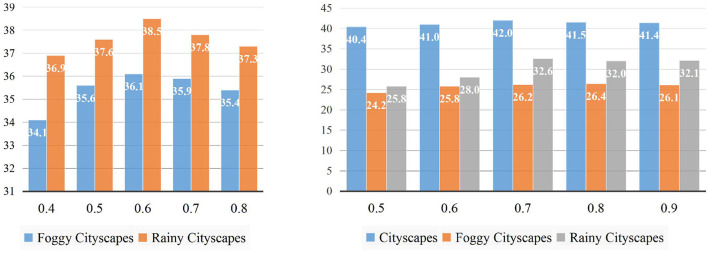
The mAP on all target domains with different confidence thresholds. The left belongs to the experiments “Diverse Weather” (from Cityscapes to Rainy-Cityscapes) while the right is “Virtual-to-Real” (from Sim10K to Rainy-Cityscapes). The vertical and horizontal axis represents overall performance (mAP) and different choices of confidence thresholds.

We also study the overall performance under different confidence thresholds, which affect the quality and quantity of pseudo labels. Although a high threshold can make preferable pseudo labels, it results in a reducing number of labels, which limits the learning of models. On the contrary, a low threshold can avoid the lack of labels, but it is easier to make mistakes, thus generating a higher proportion of false labels. Through the results from Figure 6, for the “Diverse Weather” experiments shown on the left side, the detection accuracies on the previous and the new target domains reach the highest (36.1 on Foggy-Cityscapes and 38.5 on Rainy-Cityscapes) when the confidence threshold is 0.6. As the increase or decrease of the confidence threshold (from 0.6 to 0.8 or from 0.6 to 0.4), the mAP on two domains simultaneously drops. As regards the “Virtual-to-Real” drawn on the right, the performance peaks when 0.7 is selected as the threshold. Compared to “Diverse Weather,” we find that this series of experiments has a lower sensitivity to the increasing confidence thresholds. With the improvement of the threshold (from 0.7 to 0.8 then to 0.9), the mAP shows a slight fall or even remains unchanged (from 42.0 to 41.5 then to 41.4 on Cityscapes, from 26.2 to 26.4 then to 26.1 on Foggy-Cityscapes, and from 32.6 to 32.0 then to 32.1 on Rainy-Cityscapes). In a contrast, the accuracies drop significantly (from 42.0 to 41.0 then to 40.4 on Cityscapes, from 26.2 to 25.8 then to 24.2 on Foggy-Cityscapes, and from 32.6 to 28.0 then to 25.8 on Rainy-Cityscapes) with the decline of confidence thresholds (from 0.7 to 0.6 then to 0.5). We think that the model in the “Virtual-to-Real” experiment generates superior detection results. They often have higher confidence scores so high thresholds will not filter out these results. To sum up, we determine to use 0.6 and 0.7 as the confidence threshold to generate pseudo labels, respectively.

### 5.5. Visual Detection Performance

**Figure 7:**
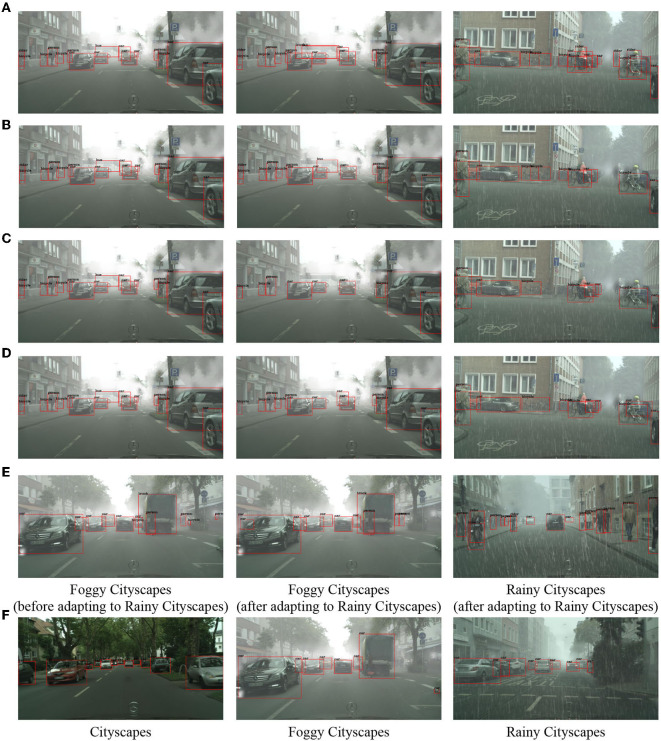
Visualized detection results on each series of experiments. The red rectangles indicate the locations of objects. **(A)** “Diverse Weather” with Methods of Section 3 (Proposed Framework). **(B)** “Diverse Weather” with Methods of Section 3 (PFT). **(C)** “Diverse Weather” with Methods of Section 3 (MIX). **(D)** “Diverse Weather” with Methods of Section 3 (FT). **(E)** “Diverse Weather” with HTCN. **(F)** “Virtual to Real” with Methods of Section 3.

Figure 7 shows some visualized detection results on experiments “Virtual-to-Real” and “Diverse Weathers.” It can be seen that our proposed framework performs well in all target domains in terms of avoiding catastrophic forgetting and transferring knowledge to new domains. Specifically, in the figure, the top and middle rows visualize detection results of “Diverse Weathers,” which are the results on Foggy-Cityscapes **before** adaptation, Foggy-Cityscapes and Rainy-Cityscapes **after** adaptation from left to right. The only difference between these two rows is the adapted methods of single-step domain adaptation, described in Section 3 and HTCN (Chen et al., 2020) respectively. Even if adapting to the new target domain (Rainy-Cityscapes), the objects on the previous target domain (Foggy-Cityscapes) remain unchanged whether big or small or suffering occlusion. At the same time, the adapted model performs well on Rainy-Cityscapes, detecting the vast majority of objects (cars, walking people, and so on). The bottom row belongs to “Virtual-to-Real” experiments including Cityscapes, Foggy-Cityscapes, and Rainy-Cityscapes, respectively. As mentioned in the Section 5.1.2, we only aim at reporting the positions of cars. The visualized results show that the model trained with our proposed domain-incremental learning framework achieves a high level of detecting the locations of cars.

## 6. Conclusion

In this article, inspired by the human brain's ability to both memorize the old knowledge and learn new knowledge, we propose a domain-incremental adaptation framework that harmonizes the discriminability and memorability for single-step domain adaptation methods when meeting a new domain. Multi-level domain adversarial training modules aim at extracting domain-invariant representations to transfer knowledge from the source domain to the current target domain. Although pseudo labels could be a link between previous-learned knowledge, the model is susceptible to be influenced by inaccurate and uncertain pseudo labels. To mitigate those negative impacts, we adopt a self-training strategy with adversarial losses, assembling the last target domain with pseudo labels together with the source domain with ground-truth labels. Our domain-incremental learning framework mainly includes two parts. The first “Recall” stage is to retrospect old knowledge from previous target domains so that it prevents memory deterioration and further refine pseudo labels. The second “Adapt” stage is to adapt and transfer from a combined source domain to the new target domain. These two stages trains iteratively to find a balance between learning and memorizing with only one labeled source domain. Experimental results have shown that our proposed domain-incremental adaptation framework performs the best compared with the existing methods.

## Data Availability Statement

The original contributions presented in the study are included in the article/supplementary material, further inquiries can be directed to the corresponding author/s.

## Author Contributions

WM coordinated its development as well as the integration of individual contributions. WL wrote the first draft of the manuscript. All authors conceptualized the structure, contributed content, perspectives, and references as well as discussed the manuscript.

## Funding

This study was supported in part by the National Natural Science Foundation of China under Grants NSFC U21A20476 and U1911401 and the Local Innovative and Research Teams Project of Guangdong Special Support Program (2019BT02X353).

## Conflict of Interest

The authors declare that the research was conducted in the absence of any commercial or financial relationships that could be construed as a potential conflict of interest.

## Publisher's Note

All claims expressed in this article are solely those of the authors and do not necessarily represent those of their affiliated organizations, or those of the publisher, the editors and the reviewers. Any product that may be evaluated in this article, or claim that may be made by its manufacturer, is not guaranteed or endorsed by the publisher.
